# Supplementary material for: Physicochemical Properties of 3D-Printed Polylactic Acid/Hydroxyapatite Scaffolds
Source: Polymers (Basel). 2023 Jun 28;15(13):2849. doi: 10.3390/polym15132849 (PMC10346171; doi:10.3390/polym15132849)
Supplement: Supplementary file 1 [file polymers-15-02849-s001.zip › polymers-2441538-supplementary.pdf]

Table S1. Volume of pores, in quantitative data obtained by micro-CT processing of images, of the 3D printed PLA/HA scaffolds with increasing infill density and/or HA contribution.

Volume fraction	PLA0HA	PLA3HA	PLA9HA	PLA13HA
infill 60%	60.089	59.017	76.730	50.569
infill 70%	55.763	58.638	63.913	50.152
infill 80%	58.110	54.479	62.227	53.000
infill 90%	44.750	45.782	48.670	30.714
infill 100%	24.590	36.271	39.499	29.500
